# Comparative Effects of Biostimulants and Fruit Enlargement Agents on Fruit Quality in Two Kiwifruit Cultivars

**DOI:** 10.3390/plants15030444

**Published:** 2026-01-31

**Authors:** Xiaoxu Sun, Kejing Zhang, Haosen Ding, Lan Li, Hong Gu, Dawei Cheng, Ming Li, Jinyong Chen

**Affiliations:** 1 National Key Laboratory for Germplasm Innovation & Utilization of Horticultural Crops, Zhengzhou Fruit Research Institute, Chinese Academy of Agricultural Sciences, Zhengzhou 450009, China; sunxiaoxu@caas.cn (X.S.); dinghaosen1415@163.com (H.D.); lilan01@caas.cn (L.L.); guhong@caas.cn (H.G.); chengdawei@caas.cn (D.C.); liming07@caas.cn (M.L.); 2 Zhongyuan Research Center, Chinese Academy of Agricultural Sciences, Xinxiang 453000, China; 3 College of Horticulture and Plant Protection, Henan University of Science and Technology, Luoyang 471000, China; zhangkejing163@163.com

**Keywords:** biostimulants, dry matter content, PGRs, Jintao, soluble solids content, titratable acidity, Zhongmi No. 2

## Abstract

Biostimulants have been increasingly investigated as eco-friendly alternatives to synthetic fruit enlargement agents in horticulture. In this study, two commercially cultivated kiwifruit cultivars, Zhongmi No. 2 and Jintao, were used as experimental materials. Three biostimulant products with distinct functional compositions were investigated: Benefit PZ (BPZ), which is rich in potassium humate and organic nitrogen; Shengcai A (SCA), containing amino acids and trace elements; and Puluosaiting (PLST), a natural seaweed extract–based formulation rich in bioactive compounds. Their effects on fruit development and internal quality attributes were compared with those of two fruit enlargement agents, 6-benzylaminopurine (6-BA) and forchlorfenuron (CPPU). Field experiments were conducted in two orchards located in Dancheng and Xixia, Henan Province, China, and treatments were applied during early fruit development. Growth traits (longitudinal and transverse diameters, single-fruit weight and firmness) and quality indicators (Soluble Solids Content, Titratable Acidity and Dry Matter Content) were measured at commercial maturity. CPPU and 6-BA substantially increased fruit size and weight compared with the control, whereas biostimulants produced moderate improvements without excessive enlargement. Notably, biostimulant treatments consistently enhanced internal quality attributes, indicating their potential to improve fruit quality without the drawbacks of excessive enlargement. Environmental and management differences between sites may also have contributed to treatment variability. These results suggest that biostimulants can improve internal quality traits while avoiding excessive fruit enlargement, representing a promising option for sustainable kiwifruit production.

## 1. Introduction

Kiwifruit (*Actinidia* spp.) is a deciduous vine belonging to the genus Actinidia of the family Actinidiaceae. It is highly valued for its unique flavor, nutritional quality, and high vitamin C content [1]. In recent years, consumer preferences have shifted from appearance-focused standards toward comprehensive quality attributes. Accordingly, improving fruit quality has become a key research priority within kiwifruit breeding, cultivation, and orchard management systems. Among the numerous cultivated kiwifruit varieties, ‘Zhongmi No. 2’ (*Actinidia deliciosa*) and ‘Jintao’ (*Actinidia chinensis*) are highly representative and possess strong market value. Zhongmi No. 2 is an excellent green-fleshed cultivar characterized by large fruit size, distinctive flavor, high dry matter content, and good storability, showing considerable potential for industrial development. In contrast, Jintao is a typical yellow-fleshed cultivar with high sweetness, low acidity, and superior flavor quality, making it highly favored in both domestic and international markets, and it has become one of China’s major export varieties [2].

Fruit enlargement agents are a class of plant growth regulators that promote cell division. They function by stimulating fruit cell division and expansion, enhancing the competitiveness of fruit for photosynthetic assimilates, coordinating source–sink relationships, and improving organic nutritional status, thereby significantly increasing fruit yield [3]. At present, fruit enlargement agents have been officially registered for agricultural use in several countries, including China, Japan and New Zealand, and are applicable to a range of horticultural crops such as kiwifruit, grape, watermelon, and melon [4]. To increase fruit size and improve marketable yield, fruit enlargement agents such as 6-benzylaminopurine (6-BA) and forchlorfenuron (C-PPU) have been widely applied in kiwifruit production. Previous studies have shown that fruit enlargement agent application can increase kiwifruit yield by 30%~60% [5]. When applied appropriately, they may also improve certain fruit quality attributes, such as increasing fruit weight, soluble sugar content, vitamin C concentration, and total sugar content [6,7]. However, the use of such agents poses potential risks [8]; improper application may result in increased incidence of fruit deformities [9], dull skin coloration [10], premature physiological maturation [11], and elevated postharvest losses [12,13], ultimately leading to a decline in overall fruit quality. Furthermore, long-term dependence on fruit enlargement agents may disrupt the balance between reproductive and vegetative growth in kiwifruit plants, thereby reducing their stress tolerance.

Biostimulants are a class of natural products or microbial-based formulations characterized by diverse sources and complex components. Their major categories include seaweed extracts, humic substances, amino acids and peptides, beneficial microorganisms, and their metabolic products [14]. Compared with fruit enlargement agents, biostimulants exhibit higher levels of safety and environmental friendliness. Acting through multiple pathways, they participate in the regulation of various physiological and metabolic processes in plants. Biostimulants can modulate the biosynthesis and signal transduction of endogenous hormones, promote cell division and elongation, and accelerate fruit development [15]. They also regulate carbon–nitrogen metabolism and the allocation of photosynthetic assimilates, thereby improving energy-use efficiency in plants [16]. In addition, biostimulants can enhance root system architecture and activity, promote root hair development, and maintain rhizosphere microbial stability, consequently improving the uptake efficiency of major soil nutrients (N, P, and K) as well as micronutrients (Fe, Zn, Mn, etc.) [17]. Meanwhile, their bioactive components can induce the expression of defense-related enzymes (e.g., peroxidase, superoxide dismutase, phenylalanine ammonia-lyase), enhance antioxidant system activity, strengthen tolerance to abiotic and biotic stresses such as drought, salinity–alkalinity, low temperature, and pest or pathogen attack, and maintain cellular membrane integrity, ultimately supporting normal plant growth and fruit development under adverse environmental conditions [18,19]. More importantly, biostimulants can facilitate fruit enlargement and quality formation without causing excessive accumulation of plant hormones or metabolic disorders, enabling improvements in fruit weight, sugar–acid ratio, and vitamin C content while maintaining desirable fruit appearance and storability [20]. Accordingly, compared with conventional fruit enlargement agents, biostimulants not only improve yield but also enhance ecological safety and fruit quality, making them an ideal approach for achieving high-quality fruit production with reduced chemical inputs. In this article, three biostimulants products with distinct functional compositions were investigated: Benefit PZ (BPZ), which is rich in potassium humate and organic nitrogen; Shengcai A (SCA), containing amino acids and trace elements; and Puluosaiting (PLST), a natural seaweed extract–based formulation rich in bioactive compounds. They represent three typical biostimulant categories, allowing a component-oriented comparison of their effects on fruit growth and quality.

Therefore, this study aims to systematically compare the effects of three types of biostimulants (BPZ, SCA, and PLST) with two commonly used fruit enlargement agents (6-BA and CPPU) on fruit growth and quality in two widely cultivated kiwifruit cultivars, Zhongmi No. 2 and Jintao, so as to explore the feasibility of replacing fruit enlargement agents with biostimulants and elucidate their mechanisms of action on kiwifruit growth and fruit quality formation [21]. Reducing the use of fruit enlargement agents and promoting the application of biostimulants can not only lower input costs but also improve fruit quality, thereby providing new technical support for the sustainable development of the kiwifruit industry [22].

## 2. Results

The experiment compared the effects of different biostimulants and fruit enlargement agent treatments on the external morphology and internal quality of two kiwifruit varieties, Zhongmi No. 2 and Jintao. Each variety was tested at two stages: commercial maturity stage and edible maturity stage. When the soluble solids content (SSC) of the kiwifruit reaches 6~9% and its firmness ranges from 12 to 16 N, the fruit is considered commercially mature. When the SSC increases to 12~18% and the firmness significantly decreases to 5~8 N, indicating that the fruit has softened and ripened, this stage is referred to as edible maturity in this study. The experimental results showed that different treatments had a significant impact on the size and quality of the kiwifruit.

### 2.1. Effects of Different Treatments on Zhongmi No. 2

#### 2.1.1. Effects on External Morphology and Internal Quality of Kiwifruit at Commercial Maturity Stage

**Figure 1:**
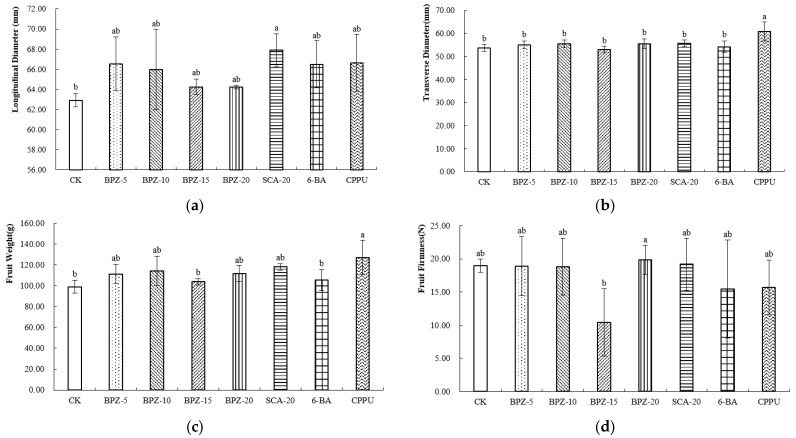
(**a**–**d**) External Morphological Parameters of Zhongmi No. 2 at Commercial Maturity Stage after Biostimulant or Fruit Enlargement Agent Treatments. Different letters above the bars indicate significant differences among treatments (*p* < 0.05) according to Tukey’s HSD test. CK, control; BPZ-5, BPZ-10, BPZ-15 and BPZ-20, Benefit PZ applied at 5, 10, 15 and 20 mL·5 L^−1^, respectively; SCA-20, Shengcai A at 20 mL·5 L^−1^; 6-BA, 6-benzylaminopurine; CPPU, forchlorfenuron.

At the commercial maturity stage, all treatments increased the longitudinal diameter of Zhongmi No. 2 kiwifruit compared with the control, and the fruits treated with SCA-20 exhibited the greatest longitudinal diameter (Figure 1a). Among these treatments, CPPU resulted in the most pronounced fruit enlargement, characterized by a longer longitudinal diameter, the greatest transverse diameter (Figure 1b), and the highest single-fruit weight. BPZ-5, BPZ-10, BPZ-20 and SCA-20 significantly increased fruit weight relative to BPZ-15, 6-BA and the control, indicating a positive effect of these biostimulants at harvest (Figure 1c). In contrast, BPZ-15 resulted in the lowest fruit firmness, which was significantly lower than that in all other treatments, while the remaining treatments showed comparable firmness levels (Figure 1d).

Different treatments also exerted varying effects on the internal quality. At the commercial maturity stage of Zhongmi No. 2, the dry matter (DM) content was the highest under the SCA-20 treatment (Table 1), whereas CPPU resulted in the lowest value. The soluble solids content (SSC) of all samples ranged from 5.21% to 15.1%, with BPZ-15 showing the highest SSC, which was significantly greater than that of the other treatments. The titratable acidity (TA) ranged from 0.88% to 2.29%; BPZ-15 exhibited the lowest TA, while CK showed the highest value, followed by SCA-20, with only a small difference between the two. Consequently, BPZ-15 presented the highest SSC/TA ratio.

#### 2.1.2. Effects on External Morphology and Internal Quality of Kiwifruit at Edible Maturity Stage

**Figure 2:**
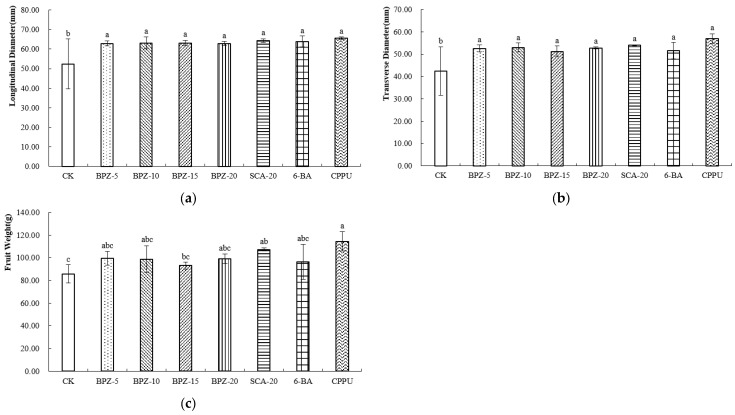
(**a**–**c**) External Morphological Parameters of Zhongmi No. 2 at Edible Maturity Stage after Biostimulant or Fruit Enlargement Agent Treatments. Different letters in the same column indicate significant differences (*p* < 0.05).

After postharvest ripening, the overall trends in fruit size remained consistent with those observed at harvest. CPPU-treated fruits retained the highest longitudinal (Figure 2a) and transverse diameters (Figure 2b) and remained the heaviest among all treatments. BPZ-5, BPZ-10, BPZ-20 and SCA-20 continued to exhibit higher fruit weight than BPZ-15, 6-BA and the control (Figure 2c). These results demonstrate that both CPPU and the more effective biostimulant treatments sustained their effects on fruit size through to the edible-ripe stage.

At the edible maturity stage of Zhongmi No. 2, the dry matter content was the highest under the SCA-20 treatment (Table 2), whereas BPZ-15 showed the lowest value. The soluble solids content (SSC) of all samples ranged from 11.79% to 15.01%. Among them, BPZ-10 exhibited the highest SSC, significantly higher than that of CK, followed by SCA-20 and BPZ-20. No significant differences were observed among BPZ-5, BPZ-15, 6-BA, and CK. The titratable acidity (TA) of all samples ranged from 0.53% to 1.29%, with BPZ-15 presenting the highest TA and BPZ-5 the lowest.

### 2.2. Effects of Different Treatments on Jintao

#### 2.2.1. Effects on External Morphology and Internal Quality of Kiwifruit at Commercial Maturity Stage

**Figure 3:**
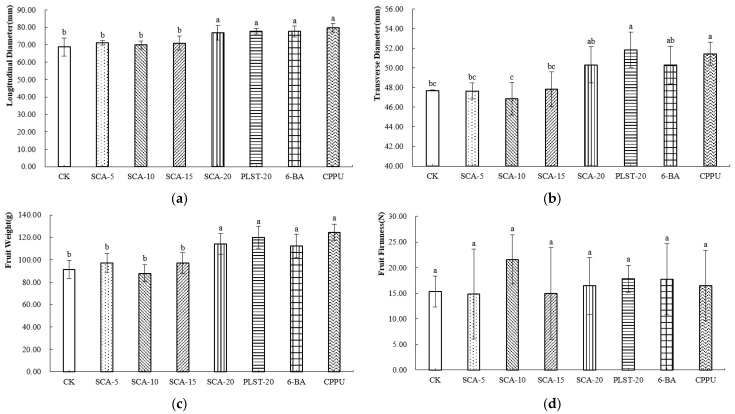
(**a**–**d**) External Morphological Parameters of Jintao at Commercial Maturity Stage after Biostimulant or Fruit Enlargement Agent Treatments. Different letters in the same column indicate significant differences (*p* < 0.05). CK, control; SCA-5, SCA-10, SCA-15 and SCA-20, Shengcai A applied at 5, 10, 15 and 20 mL·5 L^−1^, respectively; PLST-20, Puluosaiting at 20 mL·5 L^−1^; 6-BA, 6-benzylaminopurine; CPPU, forchlorfenuron.

At the commercial maturity stage of Jintao, all treatments increased fruit longitudinal diameter compared with the control, with CPPU producing the greatest enhancement (Figure 3a). For transverse diameter, CPPU and PLST-20 achieved the greatest enlargement (Figure 3b). Fruit weight exhibited a similar trend. CPPU produced the heaviest fruits, followed by PLST-20. Among the SCA concentrations, SCA-20 resulted in a higher fruit weight than SCA-5, SCA-10, and SCA-15, suggesting that the response of Jintao fruit weight increased with application rate (Figure 3c). The fruit hardness under the SCA-10 treatment was the highest (Figure 3d), indicating that this treatment condition may enhance the cell wall structure of the fruit and thereby improve its mechanical strength.

At the commercial maturity stage of Jintao, the highest dry matter (DM) content was observed under the CPPU treatment (Table 3), whereas SCA-15 resulted in the lowest value. The soluble solids content (SSC) of all samples ranged from 12.08% to 15.12%, with CK showing the highest SSC. The titratable acidity (TA) ranged from 0.57% to 1.41%, with CK exhibiting the lowest TA. Consequently, CK presented the highest SSC/TA ratio.

#### 2.2.2. Effects on External Morphology and Internal Quality of Kiwifruit at Edible Maturity Stage

The data of Jintao at the edible maturity stage are shown in Table 4. The PLST-20 treatment had the greatest impact on fruit morphological traits, showing significantly superior longitudinal diameter, transverse diameter, and fruit weight compared with other treatments, reaching 75.78 ± 2.24 mm, 47.94 ± 1.74 mm, and 110.47 ± 14.2 g, respectively. The effect of CPPU ranked second only to that of PLST-20. In addition, the differences among SCA-5, SCA-10, and SCA-15 treatments were relatively small.

At the edible maturity stage of Jintao, the dry matter (DM) content was lowest under the SCA-20 treatment and highest under the CPPU treatment. The soluble solids content (SSC) ranged from 8.8% to 14.25%, with CK exhibiting the highest value and SCA-15 the lowest. The titratable acidity (TA) ranged from 0.92% to 1.86%, with CPPU showing the lowest TA and SCA-20 the highest.

## 3. Discussion

Fruit enlargement agents such as 6-benzylaminopurine (6-BA) and forchlorfenuron (CPPU) are widely used in horticultural production. They promote cell division and expansion, thereby increasing fruit size and weight. In this article, both regulators effectively enhanced fruit size and weight; however, CPPU application was frequently accompanied by a reduction in soluble solids content (SSC), an elevated respiration rate and accelerated consumption of nutrients and water during the later stages of ripening [23]. While CPPU promotes tissue expansion, it may simultaneously suppress the accumulation and translocation of sugars within the fruit, thereby reducing the soluble sugars available for flavor compound formation. In addition, the increase in fruit volume raises metabolic demand and respiratory intensity [24,25,26], accelerating soluble sugar degradation and ultimately resulting in lower SSC during ripening. These findings suggest that although 6-BA and CPPU are effective in promoting fruit growth, this improvement may come at the cost of reduced flavor quality and diminished storage stability [27]. Thus, their application may involve trade-offs between fruit size and quality attributes.

In addition to quality-related concerns, the application of synthetic fruit enlargement agents has also raised issues related to food safety and use regulation. Although 6-benzylaminopurine (6-BA) and forchlorfenuron (CPPU) are registered for use in several fruit crops, inappropriate application or excessive dosages may result in residue accumulation and shortened pre-harvest intervals [28]. Previous studies have reported that such risks limit their practical application and require strict management in commercial orchards. These considerations further highlight the necessity of exploring alternative products that can promote fruit growth with lower safety risks [29].

For the biostimulant treatments, this study revealed substantial differences in fruit responses depending on product type and application concentration. It should be noted that the present study did not aim to isolate the effects of individual bioactive components, but rather to compare representative commercial biostimulant products with synthetic fruit enlargement agents under practical orchard conditions.

Compared with synthetic regulators, BPZ treatment showed a more moderate effect on fruit enlargement while significantly improving internal quality parameters. BPZ is mainly composed of humic substances and organic nitrogen, which are known to enhance nutrient uptake efficiency and carbon assimilation. Humic substances can promote photosynthetic activity and assimilate translocation, thereby favoring dry matter accumulation during fruit development. For example, humic acid application has been shown to improve greenhouse tomato quality, increase bacterial richness in rhizosphere soil, and enhance root growth and yield under controlled conditions, indicating practical benefits of humic-based biostimulants in vegetable crops [30]. In this study, fruits treated with BPZ, particularly at an appropriate concentration (BPZ-10), exhibited higher dry matter content and soluble solids concentration relative to the control. The improvement in fruit internal quality under BPZ treatment is therefore likely associated with enhanced source–sink relationships rather than excessive stimulation of cell expansion [31].

SCA treatment resulted in a relatively stable fruit enlargement pattern while maintaining higher fruit firmness compared with synthetic fruit enlargement agents. SCA contains amino acids and trace mineral elements, which may directly participate in metabolic regulation, enzymatic activity, and cell wall biosynthesis. Amino acids can act as both metabolic substrates and signaling molecules, whereas micronutrients serve as essential cofactors for enzymes involved in cell wall strengthening. Protein hydrolysate–based biostimulants rich in amino acids have been widely reported to enhance growth, nutrient uptake, and yield performance in horticultural and field crops, supporting the practical utility of amino acid biostimulants [32]. In the present study, SCA-treated fruits did not exhibit an excessive decline in firmness at harvest, suggesting that this biostimulant did not markedly accelerate fruit ripening. Rather, SCA treatment contributed to coordinated fruit growth while preserving textural quality.

Among the biostimulant treatments, PLST exhibited the most pronounced effect on fruit enlargement. PLST is a seaweed extract–based biostimulant rich in diverse bioactive compounds, including hormone-like substances, polysaccharides, and osmoprotectants. Seaweed extracts have been reported to stimulate cell division and expansion while enhancing plant stress tolerance. For instance, seaweed extract from *Ascophyllum nodosum* has been shown to significantly increase morphological parameters such as biomass and root and shoot growth in solanaceous crops, demonstrating the effectiveness of seaweed–based biostimulants in horticultural production [33]. In this study, PLST-treated fruits showed increased fruit size and weight compared with the control, while the reduction in fruit firmness was less severe than that observed under CPPU treatment.

It is noteworthy that orchard environmental conditions—including temperature, light availability, and humidity—directly influence the synthesis and degradation of endogenous plant hormones, thereby altering the actual effectiveness of externally applied regulators such as fruit enlargement agents and biostimulants [34,35]. The differences observed between the two orchards in CK fruit weight further highlight the influence of orchard-specific environmental conditions. Therefore, field management practices, application timing, and dosage should be carefully aligned with local climate conditions and the developmental stage of the fruit to ensure treatment stability and reproducibility.

Taken together, the present results indicate that the effects of biostimulants on fruit growth and quality are closely related to their dominant functional components. While synthetic regulators mainly promote fruit enlargement through direct hormonal stimulation, biostimulants based on humic substances, amino acids with trace elements, and seaweed-derived bioactive compounds regulate fruit development through more coordinated physiological pathways. Such component-oriented regulation may help to achieve a better balance between fruit size and quality attributes under practical production conditions.

## 4. Materials and Methods

### 4.1. Experimental Sites and Plant Material

Field experiments were conducted during the 2024 growing season in two commercial kiwifruit orchards located in Dancheng County (33.67° N, 115.19° E) and Xixia County (33.34° N, 111.34° E), Henan Province, China. Dancheng represents a low-lying alluvial plain with relatively uniform thermal conditions and intensive irrigation, whereas Xixia is located in a foothill region of the Funiu Mountains, characterized by higher elevation, larger diurnal temperature fluctuation and higher precipitation. These site-specific environmental differences may influence fruit development and treatment responsiveness.

Two commonly cultivated commercial cultivars were selected: Zhongmi No. 2 (*Actinidia deliciosa*) grown in Dancheng and Jintao (*Actinidia chinensis*) grown in Xixia. All were managed according to standard local orchard practices.

### 4.2. Treatments and Application Protocol

Three representative biostimulants with distinct functional compositions were selected: Benefit PZ (BPZ), which is rich in potassium humate and organic nitrogen; Shengcai A (SCA), containing amino acids and trace elements; and Puluosaiting (PLST), a natural seaweed extract–based formulation rich in bioactive compounds. Product compositions are provided in Table 5.

A randomized block design was used with three replications per treatment, and plants with similar vigor and fruit load were selected to minimize biological variability. For Zhongmi No. 2, the treatments included CK, BPZ-5, BPZ-10, BPZ-15, BPZ-20, SCA-20, 6-BA, and CPPU. For Jintao, the treatments consisted of CK, SCA-5, SCA-10, SCA-15, SCA-20, PLST-20, 6-BA, and CPPU.

Different concentration gradients were established for BPZ and SCA, whereas PLST was applied at a single concentration (20 mL·5 L^−1^). This concentration was selected based on preliminary experiments, which indicated that it was the most effective for both cultivars, showing enhanced efficacy and a more targeted effect on fruit enlargement. The use of a single concentration for PLST was also consistent with commercial practice, as biostimulants are typically applied according to manufacturers’ recommended dosages.

All treatments were applied by root drenching at three key phenological stages (Table 6): the early fruit stage (early May), the fruit enlargement stage (early August), and one month before harvest (mid-September). Conventional fruit enlargement agents (6-BA and CPPU) were applied by fruit dipping according to standard horticultural practices. The control group (CK) received no chemical treatment and was irrigated with an equal volume of water.

After the kiwifruits reached the commercial maturity standard, at least 30 uniform and disease-free fruits were collected for each treatment, and five fruits were randomly selected from each group for immediate measurements, while the remaining 25 fruits were left for assessment during the edible maturity stage. Morphological traits, including longitudinal diameter, transverse diameter, single-fruit weight, and firmness, were measured, along with internal quality attributes such as dry matter (DM) content, soluble solids content (SSC), and titratable acidity (TA).

### 4.3. Measurement of Fruit Growth Traits

Fruit dimensions were measured by a digital Vernier caliper (±0.01 mm), while the fruit weight was measured by an electronic analytical balance (±0.01 g). Fruit firmness was measured by a digital fruit penetrometer (TP-GY-4, Zhejiang Top Yunnong Technology Co., Ltd., Hangzhou, China) and values were expressed in Newtons (N).

### 4.4. Determination of Fruit Quality Indicators

Following standardized sampling and homogenization procedures, soluble solids content (SSC) and titratable acidity (TA) were measured by a sugar-acid meter (PAL-BX/ACID8, ATAGO Co., Ltd., Tokyo, Japan), with both expressed as percentages (%). Fruit slices approximately 2 mm thick, with peel, were taken from the equatorial region of the kiwifruit, and then dried to constant weight in a 65 °C drying oven. The dry matter content (DM) was calculated as the ratio of dry weight to fresh weight.

### 4.5. Statistical Analysis

Data were organized and graphed by Microsoft Excel. Statistical analyses were performed using R software (version 4.5.1). Statistical comparisons among treatments were performed by variance (VNOVA) with significance declared at *p* < 0.05. Results are expressed as means ± standard error (SE).

## 5. Conclusions

This study compared the effects of fruit enlargement agents and biostimulant products with distinct functional compositions on fruit growth and quality in two kiwifruit cultivars. The results indicate that although synthetic regulators such as 6-BA and CPPU are effective in promoting fruit enlargement, their application may involve trade-offs with fruit firmness and internal quality. In contrast, biostimulants exhibited more differentiated and coordinated effects. The differential responses observed among biostimulant treatments suggest that their effects on fruit growth and quality are closely associated with their dominant functional components. Humic substance–based products primarily improved internal quality attributes, and amino acid– and trace element–based products contributed to balanced fruit growth and firmness maintenance, while seaweed extract–based products showed a strong potential to enhance fruit enlargement with relatively moderate quality penalties. Overall, component-oriented selection of biostimulants may provide a more sustainable strategy to balance fruit size and quality in commercial kiwifruit production. Future studies integrating mineral nutrition, antioxidant capacity, and secondary metabolite profiles will further clarify the physiological mechanisms underlying biostimulant-mediated fruit quality improvement.

## Figures and Tables

**Table 1:** Internal Quality Parameters of Zhongmi No. 2 at Commercial Maturity Stage after Biostimulant or Fruit Enlargement Agent Treatments.

Treatment	Dry Matter Content (%)	Soluble Solids Content (%)	Titratable Acidity (%)
CK	16.82 ± 1.11 ab	5.21 ± 0.04 c	2.29 ± 0.15 a
BPZ-5	17.72 ± 1.79 ab	6.39 ± 1.16 c	1.87 ± 0.21 ab
BPZ-10	19.01 ± 1.72 a	8.29 ± 2.32 bc	1.82 ± 0.58 ab
BPZ-15	19.13 ± 2 a	15.1 ± 1.24 a	0.88 ± 0.19 c
BPZ-20	17.05 ± 0.9 ab	6.2 ± 1.11 c	2.02 ± 0.09 a
SCA-20	19.89 ± 3.55 a	6.19 ± 1.62 c	2.27 ± 0.18 a
6-BA	16.84 ± 1.06 ab	8.3 ± 2.61 bc	1.8 ± 0.43 ab
CPPU	15.61 ± 1 b	9.69 ± 1.54 b	1.33 ± 0.27 bc

Different letters in the same column indicate significant differences (*p* < 0.05).

**Table 2:** Internal Quality Parameters of Zhongmi No. 2 at Edible Maturity Stage after Biostimulant or Fruit Enlargement Agent Treatments.

Treatment	Dry Matter Content (%)	Soluble Solids Content (%)	Titratable Acidity (%)
CK	14.08 ± 8.07 ab	14.08 ± 8.07 ab	14.08 ± 8.07 ab
BPZ-5	9.8 ± 0.37 b	9.8 ± 0.37 b	9.8 ± 0.37 b
BPZ-10	19.33 ± 0.85 a	19.33 ± 0.85 a	19.33 ± 0.85 a
BPZ-15	9.39 ± 0.52 b	9.39 ± 0.52 b	9.39 ± 0.52 b
BPZ-20	13.77 ± 5.19 ab	13.77 ± 5.19 ab	13.77 ± 5.19 ab
SCA-20	19.48 ± 1.24 a	19.48 ± 1.24 a	19.48 ± 1.24 a
6-BA	10.12 ± 3.9 b	10.12 ± 3.9 b	10.12 ± 3.9 b
CPPU	13 ± 4.23 ab	13 ± 4.23 ab	13 ± 4.23 ab

Different letters in the same column indicate significant differences (*p* < 0.05).

**Table 3:** Internal Quality Parameters of Jintao at Commercial Maturity Stage after Biostimulant or Fruit Enlargement Agent Treatments.

Treatment	Dry Matter Content (%)	Soluble Solids Content (%)	Titratable Acidity (%)
CK	18.34 ± 0.56 ab	15.12 ± 0.28 a	0.57 ± 0.14 a
SCA-5	15.67 ± 2.06 bc	13.77 ± 2.77 a	0.78 ± 0.56 a
SCA-10	17.8 ± 1.52 abc	12.17 ± 1.76 a	1.08 ± 0.39 a
SCA-15	14.63 ± 3.91 c	13.7 ± 1.25 a	0.69 ± 0.46 a
SCA-20	17.97 ± 1.02 abc	13.03 ± 1.94 a	1.18 ± 0.54 a
PLST-20	17.27 ± 2.13 abc	14.3 ± 1.59 a	0.94 ± 0.58 a
6-BA	17.51 ± 1.03 abc	13.42 ± 0.78 a	0.98 ± 0.32 a
CPPU	19.29 ± 0.53 a	14.87 ± 2.84 a	1.31 ± 0.9 a

Different letters in the same column indicate significant differences (*p* < 0.05).

**Table 4:** External Morphological and Internal Quality Parameters of Jintao at Edible Maturity Stage after Biostimulant or Fruit Enlargement Agent Treatments.

Treatment	Longitudinal Diameter (mm)	Transverse Diameter (mm)	Fruit Weight (g)	Dry Matter Content (%)	Soluble Solids Content (%)	Titratable Acidity (%)
CK	63.37 ± 4.88 c	44.14 ± 3.5 bcd	69.76 ± 8.42 e	11.62 ± 0.83 a	14.25 ± 1.94 a	1.59 ± 0.4 a
SCA-5	63.89 ± 2.1 c	43.28 ± 2.61 d	72.52 ± 9.16 e	9.22 ± 1.88 a	10.78 ± 1.28 bc	1.64 ± 0.52 a
SCA-10	65.98 ± 1.71 c	44.01 ± 1.38 bcd	76.55 ± 2.99 de	11.25 ± 4.28 a	10.35 ± 2.03 bc	1.71 ± 0.6 a
SCA-15	64.9 ± 1.06 c	43.79 ± 2.53 cd	75.96 ± 8.47 de	9.39 ± 1.68 a	8.8 ± 1.39 c	1.8 ± 0.28 a
SCA-20	71.59 ± 1.86 b	46.38 ± 1.02 abc	92.68 ± 5.46 bc	8.85 ± 3.48 a	9.25 ± 0.92 c	1.86 ± 0.14 a
PLST-20	75.78 ± 2.24 a	47.94 ± 1.74 a	110.47 ± 14.2 a	11.8 ± 2.05 a	11.08 ± 2.27 bc	1.68 ± 0.38 a
6-BA	70.37 ± 2.93 b	46.09 ± 1.42 abcd	89.2 ± 9.1 cd	11.71 ± 1.78 a	10.48 ± 1.39 bc	1.62 ± 0.22 a
CPPU	75.43 ± 0.94 a	46.79 ± 0.54 ab	103.56 ± 7.2 ab	12.68 ± 2.23 a	12.05 ± 2.16 ab	1.53 ± 0.38 a

Different letters in the same column indicate significant differences (*p* < 0.05).

**Table 5:** Biostimulant Products.

Product Name	Main Components
BPZ	1% organic nitrogen + 3% urea nitrogen + 8% water-soluble potassium humate
SCA	High-purity amino acids and trace mineral elements
PLST	57.2% natural seaweed extract and plant-derived nutrient compounds

**Table 6:** Different Treatment Combinations and Application Methods.

Treatment	Dose	Remarks
CK	H_2_O	Root drenching
BPZ-5	5 mL BPZ/5 L H_2_O	Root drenching
BPZ-10	10 mL BPZ/5 L H_2_O	Root drenching
BPZ-15	15 mL BPZ/5 L H_2_O	Root drenching
BPZ-20	20 mL BPZ/5 L H_2_O	Root drenching
SCA-5	5 mL SCA/5 L H_2_O	Root drenching
SCA-10	10 mL SCA/5 L H_2_O	Root drenching
SCA-15	15 mL SCA/5 L H_2_O	Root drenching
SCA-20	20 mL SCA/5 L H_2_O	Root drenching
PLST-20	20 mL PLST/5 L H_2_O	Root drenching
6-BA	5 mg/L	Fruit dipping
CPPU	5 mg/L	Fruit dipping

## Data Availability

The data presented in this study are available upon request from the corresponding author. The data are not publicly available due to the large volume of raw data and because they are part of an ongoing study.
